# Human Dignity as Leading Principle in Public Health Ethics: A Multi-Case Analysis of 21st Century German Health Policy Decisions

**DOI:** 10.15171/ijhpm.2017.67

**Published:** 2017-06-10

**Authors:** Sebastian F. Winter, Stefan F. Winter

**Affiliations:** ^1^Faculty of Medicine, Charité - University Medicine Berlin, Berlin, Germany.; ^2^Centre for Public Health Care, Hannover Medical School, Hanover, Germany.

**Keywords:** German Health Policy, Prevention, Human Dignity, Public Health Ethics, Bioethics, Multi-Case Study

## Abstract

**Background:** There is ample evidence that since the turn of the millennium German health policy made a considerable step towards prevention and health promotion, putting the strategies of ‘personal empowerment’ and ‘settings based approach’ high on the federal government’s agenda. This phenomenon has challenged the role of ethics in health policy. Concurrently, increasing relevance of the *Concept of Human Dignity* for health and human rights has been discussed. However, a direct relationship between Human Dignity and Public Health Ethics (PHE) has surprisingly not yet been established.

**Methods:** We here conduct a systematic ethical analysis of eminent German health prevention policy case-examples between the years 2000–2016. Specifically, our analysis seeks to adapt and apply the principalism (autonomy, beneficence, justice)-based *Concept of Human Dignity* of Italian philosopher Corrado Viafora, contextualizing it with the emerging field of PHE. To further inform this health policy analysis, index databases (PubMed, Google Scholar) were searched to include relevant published and grey literature.

**Results:** We observe a systematic approach of post-millennial health policy decisions on prevention and on defined health targets in Germany, exemplified by (1) the fostering of the preparedness against pandemic infectious diseases, (2) the development and implementation of the first cancer vaccination, (3) major legal provisions on non-smokers protection in the public domain, (4) acts to strengthen long term care (LTC) as well as (5) the new German E-Health legislation. The ethical analysis of these health prevention decisions exhibits their profound ongoing impact on social justice, probing their ability to meet the underlying *Concept of Human Dignity* in order to fulfill the requirements of the principle of non-maleficence.

**Conclusion:** The observed health policy focus on prevention and health promotion has sparked new public debates about the formation of/compliance with emerging standards of PHE in Germany. We believe that the overall impact of this novel policy orientation will gradually show over mid- and long-term periods, both in terms of improvements in health system performance and concurrently in diagnostics, therapies and health outcome on individual patient level. The *Concept of Human Dignity* may soon play an even greater role in European PHE debates to come.

## Introduction

Over the last one and a half decades, prevention and health promotion have become leading public health policy topics that have been put forward high on the political agenda by many states and supranational organizations, eg, the European Union (EU)^1^ and the United Nations (UN), including the World Health Organization (WHO).^2^ Thus, public health issues and social determinants of health in Europe and at the global level have only recently appeared to become the focus of bioethical consideration.^3^ Given the financial constraints of states in economic crises and associated weaknesses in their social systems, states are increasingly aiming at sustainable policies for prevention and health promotion while striving to achieve more efficient overall health performance.^4^

These political efforts make up a constantly growing bulk of the agenda of governmental health policies, as demographic developments in many countries have resulted in a relative lack of the younger working population needed to support the states’ tax basis and statutory or private health insurance incomes.^5^ Furthermore, the gradual development of over-ageing societies in Western and Far East countries, in conjunction with a foreseeable workforce shortage in the near future, require additional measures to keep the experienced and well-trained elderly in economic production.^6^ Prevention has thus also become an increasingly high priority topic for the industry, the labor market and the economy. Consequently, in the Western world and in Far East countries, a clear tendency towards enhancement of preventive policies, supported by governments and national economies, can be observed. For Germany, the OECD Health at a Glance Europe Report shows an increased life expectancy average of 81 years (2012), and, concurrently, an activity limitation starting already around the age of 60 in both sexes.^7^ In addition, a “self reported limitation in usual activities” was observed in Germany in over a third (34,1%) of the population above 16 years of age.^7^ Thus, effective prevention of diseases appears to be more critical than ever to produce more healthy life years (HLY). It is the task of health policy to establish and promote conditions capable of reducing unnecessary suffering of a state’s citizens. In this context, and particularly over the past one and a half decades, evidence based health policy and public health ethics (PHE) have evolved as new fields of increasing scientific and sociopolitical interest.^8^

### Viafora’s Methodology for Ethical Analysis of Clinical Practice: A Role for Emerging Public Health Ethics?

Around the turn of the millennium, andparallel to the rise of novel preventive health policy approaches, Human Dignity started to become a focus of healthcare professionals, lawyers, philosophers, bioethicists and politicians.^9^ Despite this fact, the *Concept of Human Dignity* has also been disputed; some authors have even denied its usefulness in ethical guidance,^10,11^ while others have dismissed such claims as premature.^12,13^ Further research has advocated for a more differentiated use of the term.^14-17^ David Kirchhoffer argues in favor of the appropriate use of Human Dignity in ethical discourses as “the multidimensional existential reality of the human person.” This is an interesting approach, which seeks to avoid ‘dignity talk,’ ie, the unsolvable confronting reference to human dignity used as an argument by two parties of completely contrasting opinions.^14^ But also Kirchhoffer’s solution is not undisputed, as according to Matthews his view of “‘*meaningful anthropology’ where a human person ‘adequately considered’ is ‘a conscious being who possesses the capacity to knowingly and willfully act’ seems to rule out all human beings who are not meaning-seekers or able to have desires of self-worth.”*^18^ Thus, especially for these excluded human beings, *inherent* dignity plays a pivotal role in the Human Dignity concept.^18^ Notwithstanding these ethical debates, the *Concept of Human Dignity* has been embraced by international bodies, including the Council of Europe and the UN, and anchored in their international bioethical frameworks and conventions.^19,20^

Analyzing recent literature and policy events in more detail, it becomes evident that there has been some revival of the *Concept of Human Dignity* during the last decade.^21-24^ The Italian philosopher Corrado Viafora has pointed out that a – and potentially *the* – ‘locus anthropologicus’ of this millennium will lie in creating solid competencies in bioethics, including sound regulations on biomedical progress.^25^ As such, he introduced a methodology for the “ethical analysis of *clinical practice* based on the respect for human dignity,” in which he advocates for a stronger incorporation of human dignity in ethically driven clinical case management.^26^ We argue here that Viafora’s empirical, case based methodology is equally relevant and analogously applicable to public health. By applying this method to selected eminent public health case examples of the past 16 years, we wish to highlight the new role and informative potential of bioethics in German health policy and give a brief contextual outlook to the emerging field of PHE.^8^ Accordingly, this research article follows the appeal to broaden the bioethical scope^27^ by applying the case analysis method to German health policy.

Viafora’s methodology for *clinical* case evaluation is based on principalism and the respect for Human Dignity *(dignità umana)*, ie, pointing out the embeddedness and interdependency of the clinical situation in the Public Health context and state environment, including legal provisions^26^:

*“In dealing with issues of commutative and distributive justice, clinical ethics extends beyond its specific competence and steps, respectively, into the field of politics and in the field of law.”* ^26^

The *Concept of Human Dignity* as integral part of the German Constitution warrants an effort of analogously applying Viafora’s methodology to the public health and prevention case context in Germany. Human dignity is also increasingly considered important in a healthcare context.^28^ Viafora strongly believes in the “intrinsic value” of each human being, as reflected by the term human dignity. In reference to Gracia,^29^ he also considers as most crucial the ‘constructive process’ of verifying the respect for human dignity in a given case through *experience*.^26^

Viafora’s ethical methodology for clinical case evaluation can be comprehensively divided into three levels:

The first level of “intrinsic value” is based on the recognition of human dignity, ie, the unconditional-respect-generating intrinsic value of each human being. Viafora considers this idea of Gracia as *“*the ultimate criterion for distinguishing – also in the biomedical field – among moral and immoral practices.”^26^

The second level of “constructive process” is based on the four biomedical ethics principles as formulated by Beauchamp and Childress^30^: autonomy, beneficence, non-maleficence and justice. These principles, for Gracia and Viafora, are the “first moral outlines of the respect for human dignity, ie, the first ways to actualize the respect for human dignity.”^30^ Thus, their formulation and – later in the third level - relation to concrete case-examples is required in order to verify the respect we owe to the intrinsic value of human dignity. This is what is referred to by Viafora as the “constructive process” on the second level. Unlike Gracia, Viafora only uses three principles at that stage (autonomy, beneficence, justice), as he argues that the principle of non-maleficence is already included in the *Concept of Human Dignity.*^30^

The third level of “experience” consists of a “reflection phase,” ie, the necessity to “take into consideration the particular circumstances of the given case and the likely consequences of the different possible choices.” Subsequently, “experience,” on the third level “requires evaluating the outlined choice” with the particular circumstances of the given case and its likely consequences.^26^

It is common practice in bioethics to analyze individual clinical cases in terms of their ethical relevance and meaning, in order to give guidance for concrete patient treatment and the future development of a clinical field. However, these methods of individual case analysis have not yet reached broader use in the domain of public health. This research article, therefore, exhibits a health policy analysis that addresses exemplified German health prevention cases between 2000–2016. After reflection on Viafora’s human dignity and principalism (autonomy, beneficence, justice)-based method, a description of the German prevention policy structures and relevant case examples is performed, followed by an ethical evaluation that seeks to apply and adapt the *Concept of Human Dignity* to public health policy.^26^

In summary, our particular focus is to adapt and transpose Viafora’s method on human dignity to the public health context, illustrate selected German prevention policy examples, and analyze them in terms of their ethical relevance to public health with a focus on human dignity. Finally, we will address, whether - and if yes, on what grounds - the ethical and human dignity-related implications of these German prevention policy cases could advance further development of PHE.

## Methods

This research article exhibits a health policy analysis of German health prevention policy, analyzing the prevention structures and choosing eminent policy case-examples between the years 2000-2016, including a compendious ethical evaluation that seeks to apply and adapt the principalism (autonomy, beneficence, justice)-based* Concept of Human Dignity* of Italian philosopher Corrado Viafora, setting it into context with the emerging field of PHE.

Concretely, we here use a case based approach to evaluate public health policy on grounds of ethics and human dignity using the following methodology:

(1) Adapt and transpose Viafora’s *Concept of Human Dignity* to the public health context,
(2) Identify eminent examples of sustainable German health prevention policy from 2000 – 2016,
(3) Analyze implications of these health policy examples from an ethical and human dignity perspective, and
(4) Discuss the role of said implications in further development of PHE.

To further inform this health policy analysis, we have searched index databases (PubMed/MEDLINE, Google Scholar) and included relevant published and grey literature. The search was restricted to literature written in English and/or German. No formal restriction regarding the year of publication was made; however, emphasis was placed on including the most recent and updated information, whenever possible.

## Results

### The Concept of Human Dignity in Public Health – A New Foundation of Public Health Ethics?

There is ample evidence that after the millennium break German health policy made a considerate step towards prevention and health promotion, putting the strategies of ‘personal empowerment’ and ‘settings based approach’ high on the federal government’s agenda; this phenomenon has significantly challenged the role of ethics in health policy.^31-33^ The original German Social Law [Sozialgesetzgebung] by Otto von Bismarck, Chancellor of the German Reich in the 1880’s, had been based on three pillars, ie, ‘occupational health,’ ‘acute care’ and ‘prevention’; however, initially human dignity had not been a ‘direct’ aim of social policy and legislation.^34^ After being enshrined into the new German Constitution in 1949,^35^ the reference to ‘human dignity’ nowadays allows for a much more stringent application of prevention policy. However, it has taken quite a while for public health to get the attention of academic bioethics,^3,36,37^ resulting in the newly emerging field of PHE. PHE has barely been engaged with the notion and worth of human dignity and its potential usefulness to inform the foundation of ethical judgments in the public context. Despite this fact, human dignity has become increasingly interesting to healthcare professionals, lawyers, philosophers, bioethicists and politicians in general health debates.^9^ While mindful of the aforementioned partly critical ongoing debate,^10,12,16^ human dignity has gained profound importance for international legislation on human rights and biomedicine.^19,20^ This trend is also reflected by recent scientific literature as well as concrete policy events, suggesting a revival of the *Concept of Human Dignity* during the past years.^21–24^

### The Concept of Human Dignity – From Clinical Practice to Public Health

Based on our observation of these recent developments, Viafora’s “ethical analysis of *clinical* practice based on the respect for human dignity” as a bioethical concept could yield important insights when applied to examine bioethical conduct in a public health context.

Viafora’s methodological principles are derived from Beauchamp and Childress “Principles of Biomedical Ethics” from 1979,^30^ ie, autonomy, beneficence, non-maleficence, and justice, which, in turn, are founded on a contextual set of societal degrees of freedom and consented norms of society: “Such principles are derived from *considered judgments* taken from *common morality.*”^26^

To Viafora, the *Respect for Human Dignity* is a necessary premise to any principle for ethical analysis. Thus, with the principle of non-maleficence inherent to human dignity, he holds the following of Beauchamp and Childress original principles sufficient to inform ethical analysis:

(1) *Autonomy* (principle aiming at protecting and granting *responsibility*)
(2)* Beneficence* (principle aiming at protecting *integrity*)
(3)* Justice* (principle aiming at granting *reciprocity*)

From his statement made in reference to Vetlesen^38^ that for ethical clinical case judgments “moral perception is a precondition of moral performance,”^26^ it can be analogously derived that specific preconditions of a state can serve to facilitate ethical conduct in a health policy context. Thus, in addition to Viafora’s three principles, we can identify the following four preconditions in the domain of public health:

*(I)* A *profound constitutional basis* (based on *human dignity*) to enable ethico-legislative performance.

A reference to human dignity – as in the German Constitution from 1949 – has been incorporated into the 1997 Oviedo Convention of the Council of Europe^39^ and also into the Charter of Fundamental Rights of the EU in 2000,^40^ thereby establishing a common ethico-legal foundation for all 28 societies of the EU Member States. According to Michael “(human) dignity matters, because it forms the foundation of civilized society.”^41^

As such, *human dignity* understood in a public health ethical context should have

*(II)* the potential to function as a common basis for justifying legislative endeavors through ethical judgments in our pluralistic society, ie, as a *common good of societal understanding*.

*(III)* a value that fosters cultural understanding to grant citizens a *dignified life*.

*(IV)* a guarantee to the *unconditional worth* of every human being.

As a systematic ethical framework, these three principles and four preconditions, summarized in Table 1, serve to analyze the potential of the *Concept of Human Dignity* in selected public health case-examples.

**Table 1:** Summary of the Concept of Human Dignity

**Principles**		
1st Principle 2nd Principle 3rd Principle	Autonomy Beneficence Justice	→ Principle aiming at protecting and granting *responsibility* → Principle aiming at protecting *integrity* → Principle aiming at granting *reciprocity*
**Preconditions, as Based on Human Dignity**		
Precondition I Precondition II Precondition III Precondition IV	Profound constitutional basis Common good of societal understanding Dignified life Unconditional worth	→ Enable ethico-legislative performance → Common basis justifying legislation by ethical judgments in pluralistic societies → A value fostering cultural understanding for granting humane circumstances to everybody → Guaranteeing the unconditional worth for every human being in society

In approaching this endeavor, an understanding of the underlying political and cultural context that has shaped German health policy with regards to the topic of prevention and health promotion is necessary.

### From Cure to Prevention: A Paradigm Shift in German Health Policy – the 2015 German Preventive Healthcare Act

Similar to the immediate impact a physician’s therapeutic decision can have on a patient’s life, health policy-making can profoundly impact a citizen’s personal freedom and well-being.^42^ On a European level, policy decisions of such scope, for example the concerted legal provisions to prevent and contain pandemic spread of recurrent communicable diseases, is coordinated by the so-called Chief Medical Officers Meeting of the EU Member States. Health Policy in Germany - as in all EU Member States - is embedded in a framework of democratic laws of civil and social as well as penalty nature, grounded on the principles of constitutional law. Article 1 in The German Constitution *(Grundgesetz)* states the following: “Die Würde des Menschen ist unantastbar [Human Dignity is inviolable].”^35^ Given the amount of executive power attributed to State’s public health authorities – in matters dealing with life and death –, it seems pivotal to have such a profound basis for the democratic and legal framework of a country.^41^

The societal debates about a liberal or a more rigorous approach in order to reach common preventive goals can be full of tension, the reason for this dilemma is described by Hans-Martin Sass in the following phrase:

*“If health risks are present or predictable, a right to know and an obligation to tell exist. A moral not a legal obligation to follow healthcare advice applies, however; and it becomes more pressing if healthcare costs are shared in solidarity.*”^43^

Decreasing birth rates and younger retirement ages in Germany fostered the notion that a policy shift towards prevention was essential to stabilize the German social system, helping to prevent the outbreak of disease, ensure early diagnosis and facilitate improved coping strategies.^33^ Prevention was also increasingly seen to have the potential of preventing or delaying early retirement and nursing care, with specific emphasis on fostering patient participation in disease management, eg, in the sense of the recently established concept of empowering patients as “co-producers of care.”^44,45^ Therefore, German health policy had initiated the German Forum on Prevention and Health Promotion (being a voluntary, although Ministry-driven, joint venture of relevant actors in prevention) in 2002 and worked on preparations for a Law on Prevention.^33^ In addition, the Health Ministry supported initiative of “health targets in Germany” [“gesundheitsziele.de”], coordinated by the Society for Insurance Sciences & Creation [Gesellschaft für Versicherungswissenschaft und Gestaltung, GVG], evolved during the last decade, defining common goals and targets in health, quality standards, inter alia the aim of non-smoking.^46^

However, this paradigmatic shift *from cure to prevention* has taken many years of political and parliamentarian debate, culminating in the recent enactment of the German Prevention Law in July 2015.^47^ The Act to Strengthen Health Promotion and Preventive Healthcare (Preventive Healthcare Act) came to force on July 25, 2015, with the leading goal of prevention of disease before its manifestation.^47^ The Act seeks to strengthen health promotion through a direct settings-based approach, ie, intervening locally in nursery schools, primary and secondary schools, in the workplace and in nursing homes. In addition, implementation of screening tests for children, young people and adults will be reinforced and vaccination coverage fostered. A targeted cooperation between all key players in prevention and health promotion is foreseen, especially between statutory health, statutory pension, statutory accident, statutory long-term-care insurance as well as private health insurance. Through a National Prevention Conference, where participants include the social security institutions, the Federal Government, the Laender, the local authorities, the Federal Employment Agency and the social partners, mutual prevention and health promotion goals will be identified to develop a joint strategy.^47^ Generally, in Europe, there is a tendency of national health policies to approach Public Health issues through capacity building.^48^

A major burden of disease in Germany stems from so-called ambulatory care sensitive conditions (ACSCs), ie, chronic, lifestyle-associated diseases such as diabetes mellitus, chronic obstructive pulmonary disease, hypertension and cardiovascular disease that require continuous ambulatory therapeutic guidance as well as active disease self-management by patients.^49,50^ WHO estimates that through timely and effective provision of ambulatory care approximately 75% of hospitalizations for ACSCs are actually preventable, eg, corresponding to a fifth of all hospitalizations in Germany in the year 2012.^50^ While the WHO identifies a clear need to restructure ambulatory services towards more integrated care in Germany, it also highlights the role of secondary and tertiary prevention as key measures to ensure long-term functional ability of patients with chronic disease. One example – foreseen by the 2015 enacted German E-Health Law – is the implementation of a comprehensive IT infrastructure across levels of care, with patient access to their own E-Health data to “foster patient autonomy and stimulate patients’ health literacy, self-care and better adherence to treatment.”^50^

Whereas the old system had chiefly focused on therapy and nursing, the new political institutionalized strategy of prevention – including primary, secondary and tertiary prevention – granted the opportunity of a stronger public debate on financial issues of sustainable Public Health development. Clearly, there is a right for all citizens to benefit from scientific progress in genetics and healthcare^51^– a right, which should also be warranted for the public health sector. While the idea of a health system being merely a “repairing-system” is increasingly politically and publicly questioned, options for a more holistic, ie, preventive approach that would incorporate aspects of ethical and social justice, are more and more considered.^23,52^ Furthermore, since about 10 years, a clear trend towards a development of evidence-based prevention (EBP) can be observed.^53^ This development of a stronger focus on evidence in prevention coincides with the emerging era of PHE in Germany, that has recently also shaped the international debate in a broader context.^36,54^ Concurrently, within the last 5 years PHE discourses in Germany have intensified.^55^

### Eminent Examples of Sustainable German Health Prevention Policy From 2000-2016

Leading up to the 2015 Preventive Healthcare Act, five eminent German health prevention policy cases that preceded/accompanied the Act were identified for subsequent ethical analysis. These examples were chosen from different fields of prevention based on their specific eminent political and social relevance as well as on grounds of their distinct nature from one another. The chosen cases are summarized in Table 2.

**Table 2:** Eminent Examples of Sustainable German Health Prevention Policy From 2000-2016^a^

**Case 1**	State’s Preparedness against Pandemic Influenza	2006-2016
**Case 2**	HPV- Vaccination Program against Cervical Cancer	2007-2016
**Case 3**	Legal Provisions on Non-smokers Protection	2005-2016
**Case 4**	Acts to Strengthen Long Term Care	2013; 2016
**Case 5**	The New German E-Health Legislation	2015; 2016

^a^ Five eminent examples of sustainable German health prevention policy and their respective implementation timeframes are displayed. The examples were chosen from the period of 2000-2016, ie, preceding/accompanying the 2015 German Preventive Healthcare Act.

### Case-Example 1 – Preparedness Against Pandemic Influenza

Three pandemic outbreaks of influenza occurred during the last century (in 1918, 1957, and 1968). Those that occurred during the second half of the century took place in the era of modern virology and thus have been virologically characterized.^56^ All three have been identified by their presumed sites of origin, ie, as Spanish, Asian, and Hong Kong influenza, now known to represent 3 different antigenic subtypes of influenza A virus: H1N1, H2N2, and H3N2, respectively. Influenza management is hindered by unpredictability of outbreaks and huge variety of de novo forming subtypes. Evidently, true pandemics arise from genetic reassortment with animal influenza A viruses.^56^

In 2006, a new global public health threat appeared to arise in terms of pandemics, the so-called avian flu. Miller et al have described the urgent necessity of intense and transnationally coordinated preparation of public health systems to combat the pandemic threat, stating that “the evidence of multiple waves in the 20th-century pandemics underlines the importance of active real-time viral surveillance on a global scale.”^57^ Consequently, transnational collaborations are considered crucial to effective exchange of genomic, clinical, and epidemiologic data leading to the development of vaccines and treatment protocols and the identification of population-based strategies.^57^ What is striking - and has mainly been known by a select few experts in the epidemiological field - is that a timely preventive preparation of healthcare systems can be effective in saving dozens of thousands of lives. Avoiding this “hidden death threat” is first and foremost a task of establishing a sustainable health prevention policy that cares for its citizens on a population basis and *on grounds of human dignity*, ie, aiming at protecting the life of each individual citizen.

Apart from effective preparations for scaling up and implementing immunization strategies as early as possible, additional general public health measures, like obedience of hygiene protocols and certain quarantine provisions, are proven feasible strategies to reduce the course and spread of pandemic disease. One of the globally most discussed additional measures of precaution is the stockpiling of anti-viral agents and secondary antibiotics. Especially the precautious necessity of stockpiling of anti-viral agents had been the focus of Germany’s public debates. Accordingly, ethical issues and challenges associated with the pandemic threat had been considered, too. Specifically, from a political perspective, it became increasingly important to address ethical implications of pandemic influenza preparedness and to take ethical reasoning into account when making political decisions pertinent to the management of pandemics.^37^ As huge allocation decisions were to be made by politicians on State levels, the matter of social justice and proportionality had to be closely examined and carefully balanced against potential “losses” in terms of population disease burden and estimated casualties.

### Case-Example 2 - HPV-Vaccination Program against Cervical Cancer

At the beginning of the new millennium, the former director of the German Cancer Research Center (DKFZ), Harald zur Hausen, suggested to use the results of human papilloma virus (HPV) research that had identified the oncogenic potential of HPV, to develop vaccines and a subsequent strategy for systematic vaccinations against cervical cancer^58^:

*“An important practical impact originates from the development of prophylactic and therapeutic vaccines. Theoretically, cervical cancer, which contributes to approximately 12% of the global cancer burden in women, should be preventable.”* ^58^

These strategies led to the development of the first cancer vaccine and to a nationwide prevention program for vaccination against cervical cancer in Germany and other countries. Highlighting the extraordinary scientific, medical, and social impact of his work, Zur Hausen received the Nobel Prize in 2008 for “his discovery of HPVs causing cervical cancer.”^59^ A high impact on public health by the broad introduction of this cancer vaccination is foreseen. As more than 20% of the global cancer burden is estimated to be linked to infectious agents,^60^ other vaccines against many different infection-associated forms of cancer will likely be developed in the future. Moreover, rapid advances in cancer immunotherapy research support the notion that vaccine-induced immune cell reprogramming, such as via targeted delivery of RNA-encoded cancer antigens to dendritic immune cells,^61^ may soon become an important therapeutic pillar with potentially universal application in cancer treatment.

The vaccination program against cervical cancer started in March 2007, Germany being among the first European countries to implement this important step for public health and cancer prevention.^62^ HPV vaccination for the prevention of cervical cancer has since been recommended in most countries of Western Europe. Varying conditions among healthcare systems and nationwide data in the individual European countries lead to slightly different cervical cancer prevention strategies. However, there is overall consensus to monitor the public health impact of HPV vaccines.^62^

Public debates about the implementation of anti-cancer vaccines have addressed the safety and efficacy of the vaccines as well as questions regarding the optimal target group, including gender aspects. In terms of monitoring the vaccination program, a consistent epidemiologic survey about the acceptance and the factual immunologic status of the population is essential.^63^ Interestingly enough, there is a high demand for additional vaccination campaigns, in order to increase the acceptance and vaccination status also in girls with lower education backgrounds.^63^

Generally, in order to overcome a certain monitoring deficit, an health technology assessment (HTA)-report on cervical cancer has recommended the implementation of an organized screening program for quality-controlled introduction of HPV-screening and -vaccination with continued systematic outcome evaluation.^64^

### Case-Example 3 - Legal Provisions on Non-Smokers Protection

Consumption of tobacco is a risk factor for six out of eight major causes for death; in industrial nations, it is the most important health risk and considered to be the leading cause of early death.^65^

Despite these overwhelming epidemiological facts it has taken a considerate amount of time, until the Federal Republic of Germany took sufficient initiative to combat tobacco consumption in a sustainable manner. In part, this has been a matter of constitutional structure – the 16 Federal States of Germany each bearing individual legislative responsibility for Health Protection [Gesundheitsschutz] on a Laender level– such that there was a discussion about legislative competencies concerning potential legal provisions on non-smokers protection. Mons and Pötschke-Langer note the following:

“*Structural measures of tobacco prevention are effective and cost-efficient measures to reduce tobacco consumption and the related health and economic consequences. However, Germany has been very reluctant in implementing tobacco control laws for several decades. Only recently has Germany increased its efforts in tobacco control, which has resulted in a decrease of tobacco consumption and in a decrease of smoking rates, especially in youths.... For decades, politics gave in to pressure and influence of lobbyists of the strong tobacco industry, which deceived the public and politics for their economic interests and in order to establish a high social acceptance of smoking. In addition, there is the phenomenon of ‘denialism,’ which means the convinced denial of scientific findings regarding smoking and smoking prevention in opponents of tobacco control, who are not directly affiliated with the tobacco industry.”^66^*

There are certainly also other historical aspects to name in the German context. Due to the State imposed ideas of *“Volksgesundheit”* and their misuse during the dictatorship from 1933-1945, the Public Health development in Germany had almost been completely discredited in the second half of the last century and left the Germans (quite understandably) with a vague feeling of uneasiness towards all sorts of Public Health measures that would entail any kind of coercion.^67^ This historical predisposition, in fact, harbored an easy ground for the cited phenomenon of “denialism,”^68^ which has been and is still playing a significant role the debate of non-smokers protection.

Evidently, denialism has widely dominated this political debate; however, the first publicly convincing argument, which could not so easily be swept away by “liberal” deniers, was a thorough statistical meta-analysis concluding that a concrete figure of n = 3301 deaths per year in Germany were caused by passive smoking alone.^69^ The fact that almost 10 *innocent* persons die day-to-day in Germany as a consequence of passive smoking introduced a “public moral awareness” of the former merely ‘hidden’ statistics on smoking and health threats. Although produced by a complicated statistical analysis, the opportunity to imagine and being able to *relate* a concrete number of persons dying for non-self-caused reasons (eg, the threat to the pregnant waitress in a smokers bar, who has to earn money for herself and her future child, and is thus financially tied to continue working in a high health risk setting), did not only help steer the German political debate towards a more preventive health protection policy in the field of passive smoking. Critically, it also evoked a judicial constitutional court decision on anti-smoking legislation, namely the dissenting vote of German Constitutional Judge Masing. In its justification, the vote clearly indicated the legal option for the State to prohibit smoking in public areas.^70^ This ruling thus granted policy-makers and the legislator to include the argument for third party protection in further anti-smoking legislation.

*“However, the prevention of addiction is a legitimate goal of the legislator, who can allow for impeding, limiting or reducing partly from the public perception those liberally granted habits, that have at the same time the potential for addiction. The legislator can, in so far, in view to negative consequences for third parties or the public or also immediately for reduction of addiction, release legal provisions for the protection of temptations of those affected and thus for themselves”* (translation by authors)*.*^70^

It would exceed the scope of this research article to outline the various attempts of the 16 States of Germany to achieve a suitable regulation on non-smokers protection. It can, however, be exemplified with the case of the State of Bavaria, which after initially choosing a relatively loose legal provision for protection of non-smokers in public places in 2007, had to revise its legislation following a public opinion poll in 2010 that showed a 61% public support vote for a more rigorous anti-smoking policy, thus leading to a strict anti-smoking law in all public places with almost no exceptions.^71^ Kohler and Minkner recently summarized the complex situational interplay between anti-smoking legislation and direct democracy initiatives on smoking bans in Germany, exhibiting that the federal government structure and direct democratic participation in anti-smoking legislation in Germany has produced a diversity of local smoking bans and exemptions.^72^

### Case Example 4 – Strengthening Long Term Care in Germany

In response to the rapidly aging German society and concurrently increasing prevalence of the long term care (LTC) services dependent multimorbid patient population in Germany,^7^ the 2013 LTC Realignment Act [Pflege- Neuausrichtungsgesetz] brought a preventative angle into German LTC provision, specifically by fostering the 2008 “rehabilitation before care principle” and by strengthening ambulatory LTC services through establishing novel forms of residential care provision [Pflege-WGs].^73^ Notably, care benefits for dementia patients were introduced through a fundamental redefinition of the “need-for-care principle” [Pflegebedürftigkeitsbegriff] that now recognizes dementia as a mental disability warranting long-term care.^74^ Other measures included increasing the support for informal care providers, boosting flexibility of LTC services and benefits provided, and fostering intersectoral coordination between different LTC providers.^73^

The LTC Realignment Act laid the foundation of the subsequent 2015 LTC Strengthening Acts. While the First Act to Strengthen LTC [Erstes Pflegestärkungsgesetz] increased LTC insurance (LTCI) benefits by 4% and boosted benefits and services for LTC at home,^75^ measures of the Second Act to Strengthen LTC [Zweites Pflegestärkungsgesetz] are implemented implemented by 2017 and focus on supporting patient self-reliance, henceforth determining levels of care-dependency chiefly through assessment of patient self-reliance restrictions.^76,77^ Cumulatively, the proposed budget for LTC will increase by almost €5 billion by 2017, thus boosting overall benefits of LTCI services by 20%.^75^

### Case Example 5 – The New German E-Health Legislation

­­Whereas the medical and economic potential of telemedicine and E-Health for public health is enormous (a fact, mutually agreed upon by the majority of stakeholders), a practical gap towards health system-wide implementation of the necessary steps to reach the full range of the benefit for patients and healthcare professionals still exists. Thus, in Germany it has taken a relatively long period of time to gather all key players of the national healthcare system to pave the way for a concerted action towards a joint German telematics infrastructure (TI). Based on German Social Law, in 2004 a competent national authority, gematik GmbH, was established by the Federal Government, in which doctors, dentists, pharmacists, hospitals on one hand (care providers) and the federal organization of statutory health insurances on the other hand (payers) govern the TI-implementation process in a joint effort. They have made considerable progress in their common aim to build a TI (especially throughout the last three years) led by the following reasoning on the future benefits of the electronic health card (eHC). The full implementation of the eHC amongst all statutory insured citizens is considered the first milestone to Germany’s TI. As such, doctors, dentists, pharmacists, hospitals, 113 statutory health insurance companies and 70 million publicly insured will potentially be able to benefit from a well-integrated and established TI. The following specified goals highlight this process: improvement of the quality, transparency and efficiency of treatment through an electronically networked healthcare system; assurance that the rights and data of patients are protected within a connected healthcare system; simplification of the information and data exchange between all parties involved, ie, overall shorter, faster and safer ways to communicate amongst different participants within the treatment process; less bureaucracy and administrative burden for benefactors and care providers.^78^

On January 1, 2016, the Act on secure digital communication and applications in the healthcare system (E-Health Act) was enacted in Germany.^79,80^ It focuses on secure digital networking, which can save lives and fosters patient empowerment. The E-Health Act provides the essential basis for qualitative improvements in terms of prevention. Together with the Federal Commissioner for Data Protection and the Federal Office for Information Security (BSI), a system providing the best possible protection for highly sensitive patient data was established and will be implemented by doctors, dentists, hospitals, pharmacists, insured patients, statutory insurances and industry within a foreseeable timeframe. Key figures of the E-Health Act include a modern core data management for the insured, medical emergency data and medication plan on eHC, drug interaction safety, digitalized patient discharge letters, electronic patient records, wider use of telemedicine and further fostering of interoperability. Once in full function, the Act will provide an eminent preventive structural framework for the healthcare system, serving to avoid medical errors while ensuring quality of care in the broadest approach possible.

### German Health Prevention Policy Analysis From an Ethics and Human Dignity Perspective

During the last decade, German health policy has, as shown above, experienced a considerable policy shift combined with structural changes that have resulted in the institutionalization of health prevention on both Federal and State levels. This is an important step to protect and grant autonomy/responsibility for eminent questions concerning population health needs. The observed process equally pursues the principle of beneficence, aiming at the individual’s integrity*,* since efforts of these concerted policy frameworks are respecting exactly this aim. This is also shown by the agenda of the platform “health targets in Germany” [gesundheitsziele.de].^46^ Likewise, the principle of justice and reciprocity is achieved, as nothing for a population of a State is as important as having the State take the preventive health needs of its population and its health protection as serious as possible.^33^ In fact, the profound constitutional basis in terms of respect of Human Dignity is given for all ethico-legislative processes by the German Constitution.^35^ Thus, in the German society the value of Human Dignity can function as a common good of societal understanding. The new German framework for fostering prevention is explicitly designed to support the citizens of Germany in their thrive for leading a dignified life concerning their health and seeks to protect them from avoidable health threats. Finally, the unconditional worth of each individual citizen is respected in the political prevention framework.

Finding the principles and preconditions of the *Concept of Human Dignity* recurrently implemented in the recent evolutionary process of preventative healthcare structure establishment in German health policy, has prompted us to apply this concept onto each of the five eminent prevention policy examples, compendiously analyzing and evaluating them on grounds of human dignity (see Table 3).

**Table 3:** Compendious Ethical Analysis of German Health Prevention Policy From a Human Dignity Perspective

**State’s Preparedness against Pandemic Influenza** 2006-2016 *Preparedness against pandemic influenza, as provided by the State’s governments and Public Health authorities, implies complex and profound health prevention interventions, especially extensive logistic planning, including the safeguarding of public life and economy.*^81^	**Principles**			
	***1. Autonomy***	***2. Beneficence***	***3. Justice***	
	**+** Pandemic preparedness planning avoids reduction of citizens to passive subjects “awaiting disease.”	**+** Safeguarded by adequate preventative measures. **+** Concurrent protection of personal integrity.	**+** Fulfilled as prevention policy seeks to protect the maximum / ideally the entire population. This was the case in the influenza preparedness plan of the Federal Government.^82^	
	**Preconditions**			
	***I Profound constitutional basis***	***II Common good of soc. understanding***	***III Dignified life***	***IV Unconditional worth***
	**+** Taking the protection of individual Human Dignity (Article 1 of the German Constitution) as a guideline, Public Health stakeholders should ensure population-wide allocation of vaccinations.	**+** On the State level, resource allocation for influenza preparedness was importantly influenced by a mutual perception that a non-interventional / passive attitude would be highly unethical.	**+** Leading a dignified life in terms of health for the individual is only possible, if States and the Federal Government use all available knowledge and resources to guarantee a maximum degree of preparedness.	**+** Only with 100% population coverage and using all available preventive measures, a State can demonstrate and guarantee that the unconditional worth of each individual citizen is fully respected.
**HPV-Vaccination Program against Cervical Cancer** 2007-2016 *Until the last decade, a vaccination against development of cancer had been an utopian imagination.*^58^ *Launching the first ever cancer vaccine (Gardasil®) in September 2006 changed that situation drastically. Shortly thereafter, German Public Health officials publicly recommended a broad vaccination program for teenage girls, announced in spring 2007.*^62^	**Principles**			
	***1. Autonomy***	***2. Beneficence***	***3. Justice***	
	**+** Fulfilled by recommendation and offering of vaccinations on a voluntary basis. **+** Inclusion of vaccination service for target groups in SHI and PHI.	**+** Fulfilled due to good evidence for efficacy of vaccination in preventing the later development of cervical cancer.^62^	**+** Offered to all eligible teenage girls without exception. **-** Concerns of including also 1) girls with prior sexual activity and 2) teenage boys as additional groups with potential anti-cancer protection.^62^
	**Preconditions**			
	***I Profound constitutional basis***	***II Common good of soc. understanding***	***III Dignified life***	***IV Unconditional worth***
	**+** The constitutional basis for protection of Human Dignity is given in the German context. **-** This might be a point for reconsidering the strategy currently chosen, because the implementation figures of vaccination in low-education-populations are only half of those in high-educated milieus.^63^	**-** If Human Dignity is perceived as a common good of societal understanding as it should, we could question the degree of social inequality in terms of gender needs as well as concerning a lack of appropriate educational-level-adapted vaccination strategies.	**+** Concerning the aspect of leading a dignified life, clearly the aim of the program contributes to that idea in avoiding a potentially deadly disease. **-** However, this is only done for a part of the relevant population.	**-** As only a part of the population is covered, the respect for the unconditional worth of every human being is only partly met by the program. **-** What in general, seems to have to be addressed (however early the experiences with this new program are) is a more sustainable implementation effort of the vaccine program and the creation of a more sufficient epidemiologic database.^64^
**Legal Provisions on Non-Smokers Protection** 2005-2016 *There is hardly a field more disputed in societies, especially in the German public, as the debate on legal provisions on non-smokers protection.*^69^ *Therefore it seems all the more astounding that – despite these heavy debates – finally a stronger political and even plebiscitary momentum has led (at least in some States of Germany, eg, Bavaria) to clear preventive and health protective legal provisions.*^71,72^	**Principles**			
	***1. Autonomy ***	***2. Beneficence***	***3. Justice***	
	**+** Fulfilled as State has taken responsibility to protect non-smokers (potential passive smokers) who simply did not have that ‘autonomy’ before in public places, ie, as they could not opt-out of the passive smoking situation. **-** 1/3 of German annual healthcare expenditure (97 billion €) is spent to cover direct and indirect costs of smoking.^83^	**+** Fulfilled by protection of potential harm from passive smoking in public spaces. **-** Not fulfilled for forced passive smokers, eg, children, in private households.	**+** Fulfilled as prohibiting smoking in public places ensures the protection of health as a human right. **+** Smokers feeling “unjustly” treated as a result of this enactment, should be aware that, whereas smoking is a deliberate lifestyle choice, individual health is a fundamental human right necessitating protection at all times. **-** With respect to the cost of smoking (see autonomy) justice is not given for statutory health insurance, due to lack of risk premium differentiation between smokers and non-smokers.	
	**Preconditions**			
	***I Profound constitutional basis***	***II Common good of soc. understanding***	***III Dignified life***	***IV Unconditional worth***
	**+** A Constitutional Judge’s dissenting opinion, grounded on the German Constitution, initiated the State’s third-party protection measures for passive smoking prevention.^70^	**+** Smoking Community’s gradually increasing awareness and understanding towards respecting the needs of non-smokers and vulnerable groups (children, elderly, the chronically ill).^84^	**+** Even smokers increasingly understand the prohibitive regulations as a “dignity protecting consequence” of the new legislations. **-** A passively smoking child in a private household is still not leading a dignified life, as the legislations only apply to public spaces.	**+** Acceptance of smoking as harmful to one’s own health, thus appreciation of one’s personal unconditional worth. **-** The unconditional worth of passive smokers (often children) in private households is neglected.
**Acts for Strengthening Long Term Care** 2013; 2016 *In response to the rapidly aging German society and concurrently increasing prevalence of the LTC (LTC) services dependent multimorbid patient population in Germany,*^7^ *the 2013 LTC Realignment Act as well as First and Second Acts to Strengthen LTC 2015- 2017 (expected) were launched.*^73^ *Through a more patient-centered approach, these Acts induced a fundamental redefinition of the “need-for-care principle”*^74^ *as well as a focus on determining levels of care-dependency chiefly through assessment of patient self-reliance restrictions.*^76,77^	**Principles**			
	***1. Autonomy ***	***2. Beneficence***	***3. Justice***	
	**+** As patients in need for LTC can genuinely be involved with losses of degrees of freedom and self-determination, the proposed social and fiscal concept of LTC is supporting the principle of autonomy.	**+** State's recognition of dementia as a mental disability warranting long-term care and consequent redefinition of the “need-for-care principle” in the 1st and 2nd Act to Strengthen LTC as a means to adapt to our rapidly ageing society is pursuing the principle of beneficence, for an ever-growing group of individuals in need for care.	**+** Warrants a more equitable access to LTC for patients with mental illness. **-** Concern regarding gender inequality, as the large majority of informal caregivers are women.^73^ **-** Risk of cultivating income-related inequalities of care provision, since a part of care-giving is intended to be paid for privately and/or is provided by families.^50,85^
	**Preconditions**			
	***I Profound constitutional basis***	***II Common good of soc. understanding***	***III Dignified life***	***IV Unconditional worth***
	**+** For the novel German LTC-Legislation, the Constitutional basis guaranteeing Human Dignity is an important precondition. **-** Several concerns (see: Justice Principle) related to income inequality aspects^50,85^ and gender inequality.^73^	**+** The need for access to care has developed to a kind of common good of societal understanding. **-** The ultimate goal of fair access and distribution of LTC has not yet been reached (see: Justice Principle). **-** Societal understanding of extent of necessary LTC measures is hampered by huge associated societal cost.	**+** The intention and directions of the novel LTC legislation aim at the goal of leading a dignified life, now also including sufferers of mental disorders. **-** Still huge individual burden of cost for LTC-dependent citizens and individual fiscal inequalities. **-** Further social and fiscal justice by additional legislation is needed to ensure a more dignified life for informal caregivers (mostly women).	**+** The redefinition of the “need for care” principle, and with it the inclusion of dementia as a mental disability warranting LTC provision, is an important step towards realizing the preconditions for the unconditional worth of every human being. **-** Full potential not yet reached (see previously mentioned concerns; Justice Principle).
**The New German E-Health Legislation** 2015; 2016 *On January 1, 2016 the Act on secure digital communication and applications in the healthcare system (E-Health Act) was enforced in Germany.*^79,80^* It focuses on secure digital networking, which can save lives and helps to empower patients. The E-Health Act provides the essential basis for qualitative improvements in terms of prevention.*	**Principles**			
	***1. Autonomy ***	***2. Beneficence***	***3. Justice***	
	**+** Telematics and new tools of E-Health have the potential to empower patients in their ability of health literacy and self-determination. **+** Informed individual decision making of patients across the care trajectory is a core feature of medical information technologies. **-** Risk for exclusion of certain age groups, ie, foreseeable lack of “tech know-how” and associated health literacy problems with elderly people. **-** Potential risk of data protection issues.	**+** Quality assurance aspects, such as avoiding redundant examinations or preventing misinformation of doctors about a patient’s condition, are undoubtedly excellent benefits for a patient. **+** Unnecessary treatment, eg, due to possible lack of sufficient information, will be avoided in an established functioning telematics infrastructure environment.	**+** Medical information technologies within a telematics infrastructure will be available for all insured, granting fair access to all necessary health information to both patients and care givers in every relevant health context.	
	**Preconditions**			
	***I Profound constitutional basis***	***II Common good of soc. understanding***	***III Dignified life***	***IV Unconditional worth***
	**+** Novel scientific developments for health treatment improvements with increasing opportunities for medical information technology applications oblige the State - on grounds of its constitutional basis of Human Dignity- to guarantee and safeguard the framework for a sound telematics infrastructure provided by the new E-Health Act.^86^	**+** After one and a half decades the political debate on the Federal level has resulted in a common good of societal understanding, which finally lead to the implementation of a legal provision for E-Health. **-** However, the law reflects only a minimum consensus, due to reservations of many healthcare stakeholders against several E-Health applications.	**+** Healthcare quality improvement with electronic tools that provide appropriate information for insured and care givers and thereby avoid medical errors adds to ensuring a more dignified life for patients. **-** With the continual extension of telematics, health literacy problems due to age or education status might hamper the approach (see: Autonomy principle).	**+** The participatory aspect of the telematics infrastructure, ie, the- in principle - just and fair distribution and accessibility for everyone, would respect the unconditional worth of every citizen. **-** The slow process of implementation delays the expectable societal, patient, and provider benefits (ethical type-2 error/ omission bias).^87^

Abbreviations: LTC, Long Term Care; PHI, private health insurance; SHI, statutory health insurance; soc. = societal Note: Five eminent examples of sustainable German health prevention policy from 2000-2016 are analyzed and evaluated based on the three principles and four preconditions identified in the Concept of Human Dignity. Favorable, ethically aligned aspects (+) as well as potential points warranting improvement from an ethical perspective (-) of each policy example are displayed.

The compendious ethical analysis (Table 3) illustrates the immanent representation of human dignity driven ethical principles displayed in all five analyzed cases.

Whereas in Case 1 (State’s Preparedness against Pandemic Influenza) we can observe a pertinent representation of and compliance with all principles and preconditions, the remainder of analyzed cases – while overall showing substantial incorporation of human dignity based ethical content – reveal different degrees of insufficient fulfillment of the *Concept of Human Dignity*. As such, Case 2 (HPV-Vaccination Program against Cervical Cancer), Case 3 (Legal Provisions on Non-Smokers Protection) and Case 4 (Acts for Strengthening LTC) contain deficits regarding the principle of justice, since their respective policies are partially prone to disadvantage subgroups of the population in Germany (see Table 3 → Justice Principle). Whereas a constitutional basis (precondition I) is anchored in these policies, the observed disadvantage is also reflected in the merely partial fulfillment of preconditions II-IV, as both the dignified life and unconditional worth aspects are not safeguarded for those subgroups, nor is a common good of societal understanding fully accomplished. In addition, the huge societal cost associated with both smoking-associated health expenditure and LTC-provision (Cases 3 and 4), respectively, challenge solidarity thereby hampering the common good of societal understanding, touching upon aspects of the justice principle.^88^

For the still ongoing policy implementations of Case 5 (The New German E-Health Legislation) we observe a generally good compliance with all three principles, however, noting a foreseeable risk of exclusion, and thus potential future disadvantages, of certain age- and education-groups for novel extended telematic applications (eg, in patient self-management)^89^ and data protection issues.^90^ For the Human Dignity-based preconditions, a potentially reduced fulfillment of the dignified life precondition (III) for said age groups as well as a still insufficient societal consensus (II) on- and slow process of policy implementation (potential ethical type-2 error/omission bias → jeopardizing the unconditional worth precondition (IV)) suggest room for improvement.

### Role Human Dignity in Further Development of Public Health Ethics: A Future Perspective

The 21st century for many healthcare systems has coincided with a stronger focus on prevention and health promotion by governments, patients and providers.^7,91^ Another important new development is the concentration on “patients’ rights.” In Germany, a patients’ rights law has recently been established,^92^ and since 2004 an ombudsman for patients’ rights has been appointed by the Federal government.^93^ All these developments point into the right direction, however, as Cohen and Ezer^94^ have stated: “As patients’ rights movements and charters grow in popularity, it is important to link patient rights back to human rights standards and processes that are grounded in international law and consensus.” Human rights standards as such provide the basis for modern states and “(human) dignity matters, because it forms the foundation of civilized society.”^41^ One of the pivotal points of human rights is the *Concept of Human Dignity*, a concept, which has been well established in ethical reflection of novel technologies, such as genetics, during the last two decades.^95^

The gradual impact of evidence-based prevention and health promotion tools on health policy legislation will be comparable to the ongoing influence of novel scientific breakthroughs in genetics or information technologies.^51,96^ Therefore, it seems only a matter of consistency that the human rights aspect, which thus far has been attributed mainly to novel technologies,^19,20^ will be extended towards the vast evolving field of Public Health.^97^ The ethics of clinical patient care involves compassion^98^; the same should apply for health policy ethics, taking human dignity into account in its decision making process. Thus, in parallel to this movement of compassion in healthcare, the current development of PHE as a separate scientific field, in conjunction with the growing sector of Public Health Law, will enable a new fruitful debate on the importance of the *Concept of Human Dignity* as a key component of human rights in public health policy.

## Conclusion

Whereas the 1990ies in Germany were still marked by a large political and public bioethical debate on new medical technologies – chiefly in the domains of biomedical research, genetics and reproductive medicine as well as resulting in the European Human Rights Legislation^39^ – the first 16 years of the 21st century with regards to health prevention policy decisions have gradually brought to public attention new important questions about the *formation of /compliance with* emerging standards of PHE. Having in its constitution an article on the inviolability of human dignity, Germany has made a considerable step towards a health policy focus of prevention and health promotion since the turn of the millennium. As the effects of these public health decisions will gradually show over mid- and long-term periods, both in terms of improvements in individual health outcome and concurrently in diagnostics or therapies on the level of the individual patient, new concepts are needed for easing ethical evaluations of public health measures. Evidence based prevention policies will increasingly emerge^99^ and along with them a stronger need for applicable bioethical standards.^54^ In addition to this, novel information technology tools allow for better direct involvement and active participatory role of patients in biomedicine and clinical research.^100^

The debate about ‘Health and Human Rights,’ which started with the turn of the millennium^101^ in succession of the completion of the Oviedo-Convention,^39^ has also highlighted the value of the concept of respect for human dignity in the bioethical debate^9^; this has already led to new perceptions and concepts in clinical bioethics.^26^

Complementary to this, human dignity, in addition to the biomedical principles, can also serve as a valuable criterion for ethical case analysis in the public health sector. Strengthening the focus on Human Dignity, ie, integration of this criterion as a genuine part of public health policy evaluation in bioethical terms would thus be a worthwhile addition to current PHE debates. Importantly, an endorsement of human rights in the public health sector should ideally be accompanied by an extension of this concept not just to state legislation and health law, but also to the broad sector of non-governmental organizations (NGOs) and healthcare professional legislation.^97^ Finally, utilizing global health innovation technology models,^102^ successful regional health policy concepts have to be aligned with policies of neighbor states^103^ and ultimately transferred to the global level.

## Acknowledgements

The authors would like to thank Professor Corrado Viafora (Bioethics Chair, Department of Molecular Medicine, University of Padova, Italy) for inspiring them to pave the way to the Concept of Human Dignity applied in this health policy analysis. Furthermore, the authors wish to acknowledge Professor Pascal Borry (Centre for Biomedical Ethics and Law, University of Leuven, Belgium) for discussing important first conceptual ideas for this project with the author of correspondence. Finally, the authors wish to thank Professor Hans-Martin Sass (The Kennedy Institute of Ethics, Georgetown University, USA) for his valuable feedback on an outline of the project.

## Ethical issues

Ethical issues do not apply, as no primary data was collected from any human subjects for the purpose of this study and data collection was chiefly based on published and grey scientific literature.

## Competing interests

Authors declare that they have no competing interests.

## Authors’ contributions

SeFW conceptualized the research methodology, conducted the research and data analysis, co-wrote and revised the manuscript. StFW designed the research project, conducted the research and data analysis, co-wrote and revised the manuscript.

## Authors’ affiliations

^1^Faculty of Medicine, Charité - University Medicine Berlin, Berlin, Germany. ^2^Centre for Public Health Care, Hannover Medical School, Hanover, Germany.

## Key messages

**Implications for policy makers**

- In times of rapid demographic change and globalization, public health and prevention strategies are increasingly seen as indispensable for effective and durable health policy solutions needed to manage unprecedented recurring national and global challenges nowadays faced by healthcare systems.
- As most major health policy decisions, particularly in cases of prevention, will affect individual citizens’ lives in complex direct and indirect ethically sensitive ways, health policy stakeholders are increasingly confronted with a duty to evaluate and justify their decisions on grounds of frameworks of public health ethics (PHE).
- It should be acknowledged that the development and application of biomedical science in society in the past has been strongly guided and fostered by biomedical ethics, chiefly on grounds of human rights and especially on human dignity.
- In modern, multicultural, often partly secularized societies human dignity has the potential to serve as most valuable ethical baseline for public health policy decisions.
- In analyzing five major public health policy decisions of the last one and a half decades in Germany, human dignity proves to be a leading parameter in PHE, a concept that should be adopted by future public health policy legislative processes.

**Implications for public**

Health policy’s major decisions, such as Acts on E-Health or Prevention, can hugely impact society, and thus should be considered and debated by the public with at least equal importance as legislative decisions pertaining to novel biomedical (so-called hot) technologies, like preimplantation genetic diagnosis or personalized nanomedicine. However, this is currently not the case for debates about public health regulations in the important field of prevention and health promotion. Thus, public societal debates on public health policy legislation and associated ethical implications should be encouraged, prompting a need for the application of common principles for well-reflected public health ethics (PHE) discourses. In our view, modern societies would greatly benefit from the Concept of Human Dignity as a common denominator to guide analysis and reflection upon critical ethical aspects in public health policy decisions. While providing a sound structural framework for ethical debate, this concept has the potential to empower individual citizens to actively participate in public ethical and political debates.
